# The fading guardian: clinical relevance of TP53 null mutation in high-grade serous ovarian cancers

**DOI:** 10.3389/fimmu.2023.1221605

**Published:** 2023-08-23

**Authors:** Chiara M. Biatta, Michele Paudice, Marco Greppi, Veronica Parrella, Alessia Parodi, Giuseppa De Luca, Gianna Maria Cerruti, Serafina Mammoliti, Cinzia Caroti, Paola Menichini, Gilberto Fronza, Silvia Pesce, Emanuela Marcenaro, Valerio G. Vellone

**Affiliations:** ^1^ Department of Surgical Sciences and Integrated Diagnostics (DISC), University of Genoa, Genoa, Italy; ^2^ Pathology University Unit, IRCCS Ospedale Policlinico S. Martino, Genoa, Italy; ^3^ Department of Experimental Medicine (DIMES), University of Genova, Genoa, Italy; ^4^ Molecular Diagnostic Unit, IRCCS Ospedale Policlinico S. Martino, Genoa, Italy; ^5^ Oncology Unit, IRCCS San Martino IST, Genoa, Italy; ^6^ Oncology University Unit, IRCCS Ospedale Policlinico San Martino, Genoa, Italy; ^7^ Mutagenesis and Cancer Prevention Unit, IRCCS Ospedale Policlinico S.Martino, Genoa, Italy; ^8^ IRCCS Ospedale Policlinico San Martino, Genova, Italy; ^9^ Pathology Unit, IRCCS Istituto Giannina Gaslini, Genoa, Italy

**Keywords:** high grade serous ovarian carcinoma, TP53, immunohistochemistry, sanger sequencing, ovarian cancer

## Abstract

**Background:**

we evaluated the concordance between immunohistochemical p53 staining and TP53 mutations in a series of HGSOC. Moreover, we searched for prognostic differences between p53 overexpression and null expression groups.

**Methods:**

patients affected by HGSOC were included. For each case p53 immunohistochemical staining and molecular assay (Sanger sequencing) were performed. Kaplan-Meier survival analyses were undertaken to determine whether the type of TP53 mutation, or p53 staining pattern influenced overall survival (OS) and progression free survival (PFS).

**Results:**

34 HGSOC were considered. All cases with a null immunohistochemical p53 expression (n=16) showed TP53 mutations (n=9 nonsense, n=4 in-frame deletion, n=2 splice, n=1 in-frame insertion). 16 out of 18 cases with p53 overexpression showed TP53 missense mutation. Follow up data were available for 33 out of 34 cases (median follow up time 15 month). We observed a significant reduction of OS in p53 null group [HR = 3.64, 95% CI 1.01-13.16].

**Conclusion:**

immunohistochemical assay is a reliable surrogate for TP53 mutations in most cases. Despite the small cohort and the limited median follow up, we can infer that HGSOC harboring p53 null mutations are a more aggressive subgroup.

## 1 Introduction

Epithelial ovarian cancer (EOC) is the fifth most common cause of female cancer death in the developed world. It affects every year 21,750 women in the USA of whom 13,940 will die of the disease (1). Despite aggressive surgeries and combined chemotherapies, the prognosis remains worrisome. The reason for this high mortality rate is the late presentation, meaning that more than 70% of EOC are diagnosed at stage III or IV with a 5-year Overall Survival of approximately 15-30%.

In recent years the therapeutic landscape for EOC has seen a revolution expected for thirty years with the introduction, the PARP-inhibitors, changing the prognosis of the BRCA mutated subgroup of EOC. In SOLO-1, which investigated Olaparib in newly diagnosed advanced BRCA mutated ovarian cancer, the 3-years risk of disease progression or death was 70% lower with Olaparib than with placebo (60% vs. 27%, HR 0.30) (2).

EOC is not a single disease. Its histopathology is heterogeneous and each EOC subtype harbors genetic mutations that are being assessed for their potential to predict the efficacy of molecularly targeted treatments (3). The most frequent subgroup, accounting for 70% of all EOCs, is high-grade serous ovarian cancer (HGSOC), which is considered to originate from serous tubal intra-epithelial carcinoma (STIC) and is characterized by mutation in TP53 gene in 95% of cases.TP53 is the most frequently mutated gene in cancer, with mutations identified in at least 50% of human malignancy. The protein p53 is a homotetrameric transcription factor with tumor suppression functions. It controls the expression of hundreds of target genes in order to maintain homeostasis and genome integrity. It can activate DNA repair proteins when DNA has sustained damage, arrest cell growth by holding the cell cycle at the G1/S transition, allowing DNA repair, and initiate apoptosis if DNA damage proves to be irreparable. It’s also involved in senescence, autophagy as well as processes that oppose oncogenic metabolic reprogramming (4).

In normal condition, wild-type p53 is maintained at low levels by the E3 ubiquitin ligase MDM2 that polyubiquitinates p53, marking it for proteasomal degradation. In response to cellular stress, several mechanisms, disrupt the MDM2-p53 association, leading to the stabilization and the activation of p53 (5).

More recent research has focused on the epigenomic control of p53 providing evidence that microRNAs and long noncoding RNAs can play a role in the epigenomic control of p53 expression. These findings suggest that epigenetic changes may be a promising target for cancer prevention and treatment (6, 7).

Over 36,000 TP53 mutations have been reported, and for this reason, it is challenging to find a drug that could be effective for all mutations. Approximately 80% of TP53 mutations are missense mutations and lead to an overexpression of mutp53 in the cells that can be promptly identified by immunohistochemistry (IHC). The other mutations as frame-shift, nonsense and splice-site mutations are collectively known as p53-null mutations and they result in the absence of an encoded protein.

In this setting it is evident how relevant the identification of molecular prognostic factors is, which could potentially identify subgroups of tumors with greater aggressiveness and which require therapeutic modulation with more aggressive treatments and close follow-ups.

Here we describe how p53-null mutations have implications in prognosis and in the aggressiveness of HGSOC and how a simple and inexpensive diagnostic tool as IHC can be used as an alternative to Sanger sequencing in discriminating between null and missense mutations of TP53.

## 2 Materials and methods

### 2.1 Tumor samples

Our cohort consisted of 36 gynecological tumors (serous ovarian, fallopian tube and peritoneal carcinomas) collected from IRCCS Ospedale Policlinico San Martino. 34 HGSOC, one low-grade serous tumor (LGSOC), and one mucinous carcinoma were included in the final analysis as study controls. 29/34 HGSOCs were at an advanced stage, classified as FIGO (International Federation of Gynecology and Obstetrics) III or IV, whereas 5/34 were at an early stage, classified as FIGO I or II.

Survival and other clinical data were available for many patients. Based on immunohistochemical analysis of p53 protein expression, we matched p53 null mutation cohort, non-otherwise selected, with p53 protein overexpressed cohort consecutive unselected with similar histopathologic features. We divided the patients into two cohorts: one characterized by p53 null mutations, and one characterized by p53 overexpression.

After histopathological HGSOC diagnosis, if deemed suitable, patients underwent primary debulking surgery (PDS) followed by six cycles of chemotherapy. If complete surgical debulking was not judged feasible, two sets of three cycles of neoadjuvant chemotherapy (NACT) interspersed by interval debulking surgery (IDS) were performed. The standard regimen of chemotherapy contains Carboplatin AUC5 plus Paclitaxel 175 mg/m^2^ every three weeks with addition of Bevacizumab or Olaparib based on BRCA status, when investigated. In case of disease recurrence, after considering secondary debulking surgery (SDS) for a subset of selected cases, patients received second line chemotherapy according to common guidelines. None of the patients had intraoperative complications. One of them was also affected by metastatic non-small cell lung cancer (NSCLC) and one already had EOC metastasis.

Progression Free Survival (PFS) was defined as time from the date of biopsy/surgery and consensual diagnosis of HGSOC to the date of progression diagnosed with imaging and laboratory techniques. Overall Survival (OS) was counted from the date of biopsy/surgery and consensual diagnosis of HGSOC to the date of death or last follow up as recorded in hospital medical records, doctors’ rooms, and publicly available death notices. Written informed consent was obtained from all subjects. All methods were carried out in accordance with the approved guidelines.

Matched formalin fixed paraffin embedded samples were obtained from our diagnostic pathology laboratory (IRCCS Ospedale Policlinico San Martino). Among the study samples, we included four kinds of specimens: 1 FNAB (Fine Needle Aspiration Biopsy), 13 LPS (laparoscopy), 18 primary debulking and 2 secondary debulking surgery (after disease relapse). This retrospective series of HGOSC specimens was prepared according to standard protocols. In brief: after the surgical excision, all the specimens were sent unfixed to the pathology units where they were fixed in 10% buffered formalin (12–18 hours); after grossing, the samples were routinely processed, and paraffin embedded to obtain histological slides stained in hematoxylin and eosin (H&E). The paraffin blocks were kept in dedicated archives, at room temperature, in cardboard boxes kept away from dust, light and heat sources.

Two pathologists [VGV, CMB] to confirm diagnosis, supported by standard immunohistochemistry biomarkers panel (Cytokeratin 7, Cytokeratin 20, Vimentin, WT1, Napsin-A, ER, PR, Ki67 and p53), histological grade and pathological stage, reviewed all tumor tissue. Following initial surgery, patients were staged according to the FIGO criteria.

Sections were ascertained from tumors for determination of percent tumor cells following H&E staining and for DNA extraction. Tumors containing at least 5% of tumor cells were selected for this study (5).

The most significant paraffin block was selected for molecular analysis according to the following criteria: optimal fixation/storage, high representativeness of the entire neoplasia, high tumor cellularity, low percentage of stroma cell, fibrosis and necrosis. From selected samples, manual macrodissection was performed and sections (three sections of 10μm thickness) were obtained for molecular analyses.

### 2.2 p53 immunohistochemistry

One tumor-rich sample per case, a 3-μm-thick section from a formalin-fixed, paraffin embedded tumor tissue block, was selected for immunohistochemical analysis. Staining was detected with the automated ultraView Universal DAB procedure on the BenchMark ULTRA IHC/ISH Staining Module, Ventana with anti-p53 (clone DO7, prediluted,Ventana, Innovation Park Dr. Tucson, AZ, USA). Ventana Medical Systems’ (Ventana) CONFIRM anti-p53 (DO-7) a mouse monoclonal antibody (IgG1, kappa) directed against human p53. The antibody is intended for laboratory use to qualitatively identify by light microscopy wild type and mutant p53 in sections of formalin-fixed, paraffin-embedded tissue on a Ventana automated slide stainer.

Nuclear staining was considered a positive reaction. The extent of nuclear staining was estimated to the nearest 5% level of positive tumor cells, reporting the actual percentage for each case.

All stains were done within one week after sectioning. Stained slides were examined by an experienced surgical pathologist [VGV, CMB, MP] who was blinded to molecular data.

The percentage of cells showing positive nuclear staining was estimated and report-ed in three categories: ≥50% positively stained nuclei (overexpression); >1% and <50% stained nuclei (partial expression); ≤1% positively stained nuclei (no expression/null) (5).

### 2.3 Sanger sequencing molecular analysis

All samples underwent Sanger sequencing using the identical DNA. PCR primers and conditions for amplifying genomic DNA sequences within exons 2-11 of TP53 gene were those recommended by the International Agency for Research on Cancer (IARC) TP53 database (https://p53.iarc.fr/Download/TP53_SangerSequencing_IARC.pdf).

Briefly, PCR products were purified with the enzyme ExoSap-IT (USB) and consequently, sequencing reaction done with BigDye. Terminator v3.1 Cycle Sequencing Kit (Applied Biosystems). Subsequently, products were purified, and sequencing was performed on 3500 Genetic Analyzer capillary sequencing instrument (Applied Biosystems).

Electropherograms were analyzed by visual inspection of sequences imported in MacVector software. Variants found were compared against the human TP53 reference genomic sequence NC_000017.10 (transcript NM_000546.5) and biological/clinical significance (if present) carried out thanks to IARC and Cosmic databases (5).

The Limit of Detection (LoD) of mutational testing by Sanger sequencing in our lab is extimated approximately 12-15%. Considering the issue of heterozygosity, the minimal neoplastic component present in the section should be quantitatively double the instrumental LoD, according to SIAPeC-IAP (Italian Society of Anatomic Pathology) recommendations. For example, a sample with 10% tumor cells should be tested with an assay with LoD of at least 5% (8).

### 2.4 Statistical analyses

Clinicopathological and laboratory data was imported in MSExcel™ spreadsheet and analyzed with dedicated statistical software MedCalc. The Chi-squared test was used to evaluate the association between categorical data while continuous values were com-pared using Kruskal-Wallis test.

Kaplan-Meier survival analyses compared by the log-rank (Mantel-Cox) test were undertaken to determine whether the type of TP53 mutation, or p53 staining pattern influenced overall (OS) and progression free survival (PFS) in HGSOCs. p<0.05 was considered statistically significant.

## 3 Results

Clinicopathological characteristics of the cohort studied are summarized in **Table 1**. The mean age at diagnosis of both cohorts was 66.3 years (range 48 – 79), being 68.8 (range 48 – 79) in p53 null group and 64.1 (range 54 – 78) in p53 overexpressed group. The slight age difference at diagnosis between the two cohorts was statistically significant. The median follow up was about 15 months (range 1 – 57), evaluated on 33 patients being one lost to follow up. All of them were high-grade serous ovarian carcinomas (HGSOCs) and one (2,9%) had a second clear cell component.

**Table 1:** Clinicopathological characteristics.

	p53 null (n=16)	p53 overexpressed (n=18)
Mean age (years)	68.8 ± 7.1	64.1 ± 7.5
Mean follow-up (months)	13.5 ± 10.2	16.8 ± 12.8
Early stage (FIGO I-II)	1	4
Advanced stage (FIGO III-IV)	15	14
NACT	5	5
No debulking surgery	3	0
Macro residual disease (MDR)	3 + 3	3
mOS(months)	13.5 ± 10.2	16.8 ± 12.8
mPFS(months)	7.4 ± 8.8	10.4 ± 9.1
Deaths	7	3

**Figure 1:**
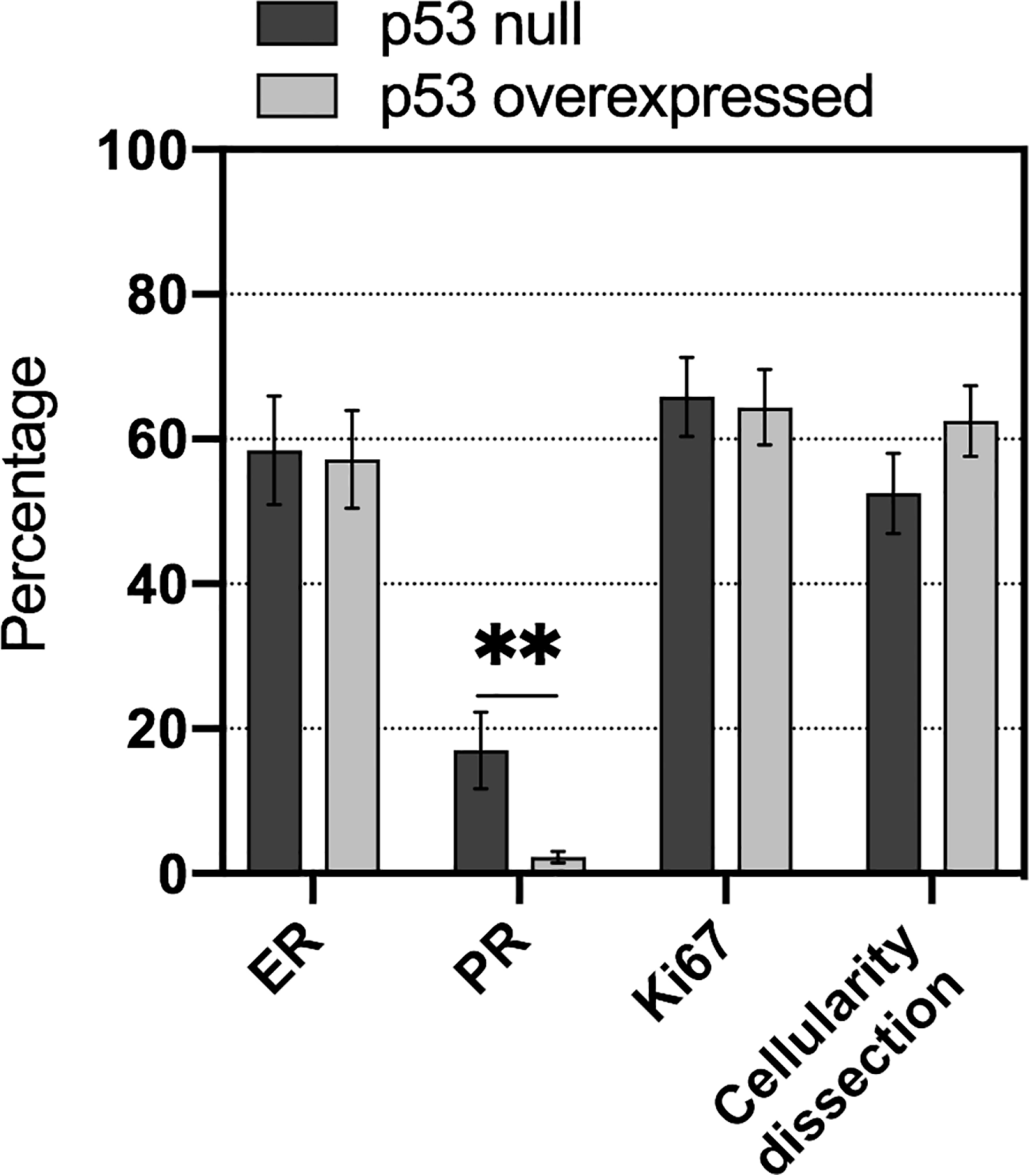
Comparison of immunohistochemistry expression of biomarkers routinely used in HGSOC diagnosis. ER, Estrogen Receptor; PR, Progesteron receptor. **p value>0.01.

The tumor cellularity (%) of the samples, sometimes obtained through macrodissection, was very high (57.8 ± 21.8), regardless of the type of specimen. No statistical difference in tumor cellularity was observed between samples belonging to p53 null and p53 overexpressed groups (**Figure 1**).

Among all biomarkers included in standard immunohistochemistry panel used in HGSOC diagnosis, only Progesterone (PR) showed statistically significant difference, being less expressed in cases belonging to p53 overexpressed cohort.

Neither ER (Estrogen) nor proliferation index Ki67 expression showed statistical significance (**Figure 1**).

**Figure 2:**
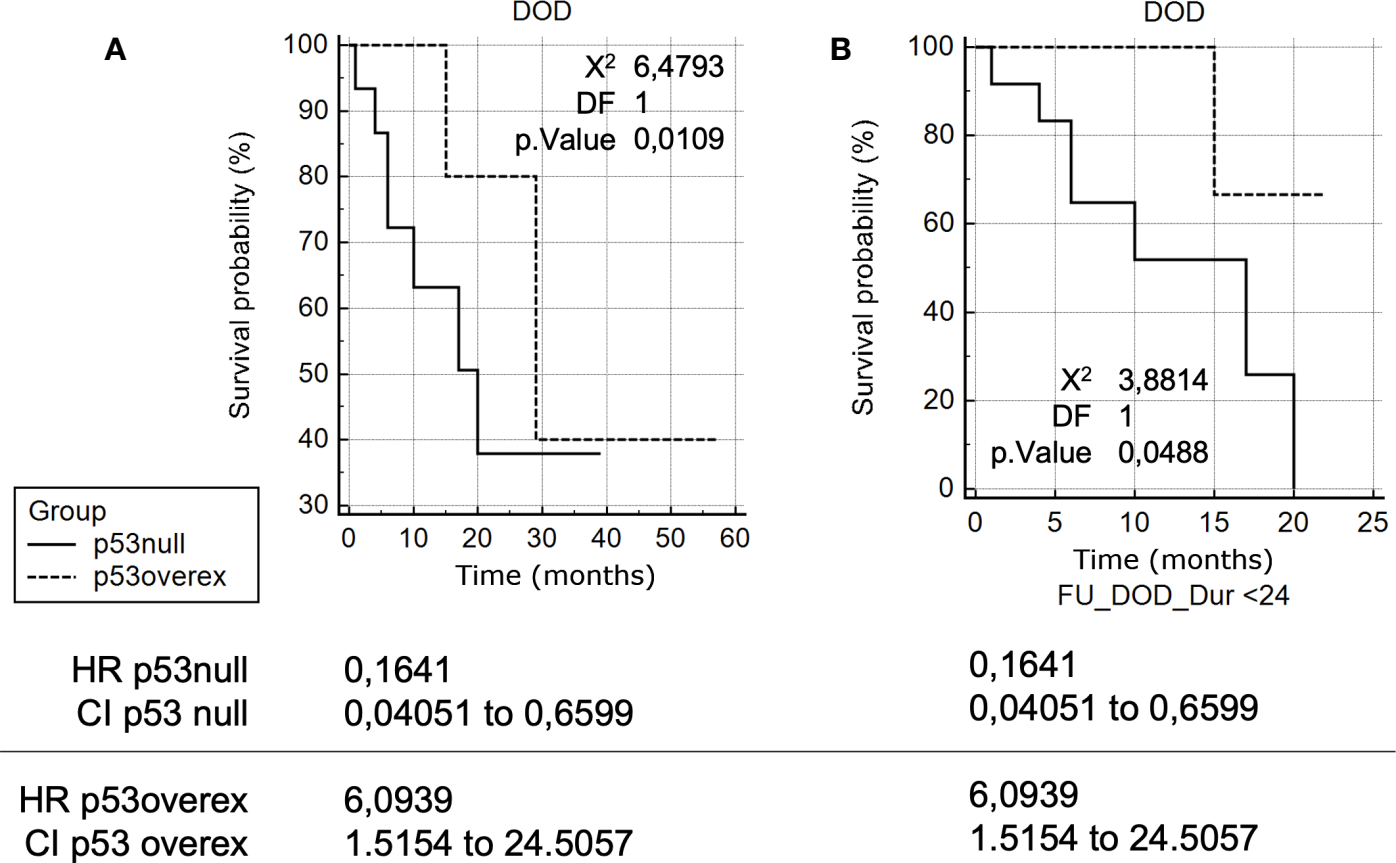
**(A)** Kaplan-Meier survival curve analyses in HGSOCs. Kaplan-Meier survival curve analysis of TP53 mutation-positive HGSOCs for Death Of the Disease (DOD), in months, of patients with p53 overexpression compared to patients with p53 null. **(B)** Kaplan-Meier survival curve analyses in HGSOCs (24 months). Kaplan-Meier survival curve analysis of TP53 mutation-positive HGSOCs for DOD.

There was a significant association of p53 null with increased risk of HGSOC-related death [HR = 3.64, 95% CI 1.01-13.16] compared with p53 overexpressed (**Figure 2A**).

Analogously, an even more significant association of p53 null with increased risk of HGSOC-related death within 24 months of follow up [HR = 6.09, 95% CI 1.52-24.51] was observed compared with p53 overexpressed (**Figure 2B**). Conversely, the difference in PFS was not statistically significant between the two groups.

All 34 HGSOCs (100%) showed aberrant p53 immunohistochemical expression, whereas the two study controls (LGSOC and mucinous carcinoma) had wild-type TP53, with p53 expression respectively 20% and 5% (**Supplementary Figure 1**).

Of 34 HGSOCs, analyzed for TP53 mutation by Sanger sequencing, 94,1% (32/34) tumors contained mutations of potential biological and clinical interests. In 16 out of 18 (88.9%) p53 overexpressed samples, we identified at least a missense mutation, in some cases associated to synonymous mutations, all occurring in the p53 DBD; of the remaining 2 (11.1%) we couldn’t find any mutation between exons 2–11 of TP53 gene in one, while the other has not been evaluated due to poor DNA quality. The p53 null immuno-labeling pattern was always associated to mutations: 4 nucleotide deletions (25%), 1 nucleotide insertion (6.3%), 2 splice-site mutations (12.5%), and 9 nonsense mutations (56.3%). Not surprisingly, all samples with nonsense mutations exhibited no p53 staining (p53 null).

p53 neutral polymorphism was not included in further analysis. By combining two immunohistochemical staining patterns (p53 null and p53 overexpression), the immunohistochemical analysis correlated with the mutational analysis in 94.1% of cases.

**Figure 3:**
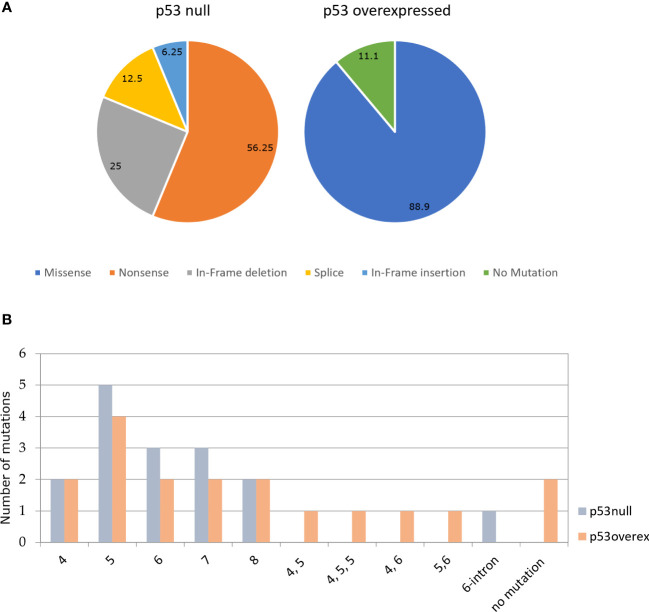
**(A)** Percentage of the different types of TP53 in p53 null (N=16) and p53 overexpressed (N=18). **(B)** Percentage of mutations in each exon of TP53 in p53 null and p53 overexpressed.

Immunohistochemical and mutational analysis data are summarized in **Figure 3A**.

The majority of TP53 mutations with p53 null immunophenotype identified in this study (81.25%; 13/16) were located in the p53 DBD, with the exception of p.Thr81fs, p.Arg306* and p.Glu51*. ‘Hot-spot’ codons for mutation included p.Arg175 (found twice in p53 overexpressed group) residing within the p53 DBD, that have previously been recognized as TP53 mutational ‘hot-spots’ in human malignancy, as well as p.Gly245Ser(**Figure 3B**). Both of these mutp53 have been recognized to be GOF mutations (9).

Most of the mutations identified in the study samples through Sanger sequencing and studied on IARC and Cosmic databases resulted pathogenic or likely pathogenic. But 4 of them had not yet been described.

## 4 Discussion

### 4.1 p53 immunohistochemistry as surrogate marker for mutational TP53 status

Individual examination of HGSOCs diagnosed according to current criteria revealed that p53 was abnormally expressed in 100% of these tumors. The definition of abnormal expression was taken from a previous study (10) in which p53 was either completely negative or overexpressed in more than 50% of tumor cell nuclei.

Notably, the frequency of abnormal expression was similar to the reported frequency of TP53 mutations, present in approximately 94% of HGSOCs. This range highlights the limitations of previous TP53 mutation studies, as the methods used were not sensitive and were not expected to detect all mutations leading to loss of expression of the functional p53 protein. Technical limitations include the inability to study only some exons, only mRNA, to assess deletions and inversions, and to sequence insufficient depth to detect somatic mutations present in mixed tumor cell and normal cell samples.

Analysis of p53 expression by immunohistochemistry is a rapid, inexpensive, and widely available method that may be useful as a surrogate marker for TP53 mutation status, but its ability to predict mutation status using both current standards for interpretation of p53 expression and comprehensive and detailed analysis of TP53 has been predicted that missense mutations in TP 53 are associated with accumulation of p53 protein in the nucleus and overexpression should indicate missense mutation. Havrilesky et al. found that 69% of TP53 mutations detected in advanced ovarian cancer were missense, and overexpression. Havrilesky et al. estimated that p53 overexpression by immunostaining (defined in their study as >30% of cells staining positive, although strong staining was seen in 75-100% of cells in most of these cases) (9) concluded ha TP53 overexpression is a fully sensitive marker for the detection of missense mutations (100%), but it is also seen in cases with other mutations (insertions, deletions, nonsense mutations) or undetectable mutations. The lack of TP53 mutations detectable by p53 overexpression (8/98,8% in their study) is problematic due to the small number of cases. It is unclear whether this lack of specificity is due to false-positive immunostaining results or false-negative mutation analysis results. Recently, it has been suggested that if immunostaining for p53 is completely negative, this should be associated with mutations undergoing non-sense degradation with high sensitivity and specificity, but there is relatively little data linking this immunostaining pattern to mutation analysis (11, 12). Yemelynova et al. reported 14 cases in which loss of p53 expression and localized expression predicted TP53 mutations from wild type with 88% sensitivity and 100% specificity (11).

In our study, the frequency of null mutations was completely consistent with case series in which we observed a complete absence of p53 expression (100%). Although this rate seems high, there may be a bias in the TP53 mutations recorded, as many studies limit their analysis to exons 5-8 and omit null mutations outside exons 5-8. Further studies with a larger number of cases are needed to determine how reliable this correlation is.

### 4.2 TP53 null mutations and unfavorable outcome

Our second finding was that HGSOCs with complete absence of p53 expression were associated with an unfavorable outcome. This confirmed an observation first made some years ago (13, 14). In particular, Shahin et al, revealed that HGSOCs harboring TP53 null mutation had an increased risk for tumor-related death [HR 2.17 (1.35–3.51)] compared with TP53 missense mutation.

We found a risk for death of similar magnitude [HR 3.63 (1.01–13.15)] for patients with no expression in p53. Limiting follow up to 24 months makes this data even more pronounced [HR 6.09 (1.52–24.51), p<0.01] suggesting that p53 null HGSOCs are a sub-group of ovarian cancer with a particularly adverse presentation.

These differences support the possibility of biological differences related to the nature of the TP53 mutation. Findings like similar frequency of macro residual disease (MRD) and similar requirement of NACT administration in both groups of our study didn’t take into account that 3 patients (~19%) belonging to p53 null group were not deemed suitable to PDS against no patient in the p53 overexpressed group. Indeed, they received first line chemotherapy instead of NACT and certainly had pelvic and abdominal disease when chemotherapy was started.

Our findings are somehow in contrast to previous meta-analyses that found that p53 overexpression is a risk factor for shorter survival in women with ovarian cancer (15). While these meta-analyses were adequately powered to show a modest effect, the authors clearly acknowledge the bias of studying different heterogeneous histologic types in terms of initial stage, outcome and response to chemotherapy (16). Furthermore, the inclusion of non-HGSOC patients without p53 abnormalities and with good prognosis hinders attempts to understand the clinical significance of different p53 expression patterns. Indeed, biomarker studies in cohorts with mixed disease types are more likely to identify type-specific (diagnostic) markers rather than type-independent prognostic markers, and the adverse outcomes identified may be related to histologic type rather than p53 overexpression.

Since these tumors usually lack p53 abnormalities, the presence of these mutations can be associated with a poor prognosis across all cancer stages because p53 alterations are associated with an unfavorable subgroup of ovarian carcinoma (HGSOC).

This controversy demonstrates the importance of examining prognostic biomarkers in homogeneous tumor cohorts.

These data are preliminary considering the small number of patients, the limited median follow up and the fact that one of the three patients not feasible for PDS was also affected by metastatic non-small cell lung cancer (NSCLC). Nevertheless, we can infer that HGSOC harboring p53 null mutations are a more aggressive subgroup, especially in its clinical presentation. This notion should lead the clinician, once more, to be as timely as possible, in the effort of not wasting the chance to eradicate a pathology that can briefly escape from the intent of cure.

In the last years we have been facing outstanding improvements in targeted therapy in the whole oncologic landscape. Regarding ovarian cancer, after years of limited therapeutic innovation, PARP inhibitors Olaparib, Niraparib, Rucaparib and Veliparib are showing important clinical benefit for patients with BRCA mutations or HRD deficiency (2, 17–19).

In this perspective, research on TP53 gene is very attractive as it is altered in 95% of HGSOCs and it is the most mutated gene in cancer.

New drugs that restore the wild-type structure and function of mutant p53 are underway and missense p53 proteins, which are found at high levels in cells due to loss of MDM2 regulation and other mechanisms, appear to be a promising drug target (20). The most promising compound is PRIMA-MET (APR246), a prodrug that after hydrolytic conversion to its active substance (Methylene Quinuclidinone) binds to cysteine in p53 and reactivates p53 wild-type functions. Nowadays there are 10 ongoing clinical trials with APR-246 and the combination of APR-246 plus Azacitidine for the treatment of MDS started phase 3 in January 2019.

Moreover, the large number of diverse p53 mutations and the notion that specific mutp53 have different forms and cellular effects, are leading to the investigation of others therapeutic strategies to selectively target specific classes of mutations including prevention of p53 degradation by MDM2/4 antagonists, disruption of aggregates of mutp53 and other selective strategies aiming to target the single specific mutation. Even for p53 null-mutations, which appear to be the most difficult mutation to restore, a combination treatment with nonsense-mediated mRNA decay (NMD) inhibitor has shown an increase in tumor cell elimination, shedding light on a field unexplored so far.

Despite the recent progress described the significance of TP53 mutations as well as the effect of specific TP53 mutations (especially GOF TP53 mutation) on EOCs are to be better understood. Only with a deeper knowledge of p53 biology it will be possible to develop targeted drugs against a critically important protein for development of HGSOCs and many others cancer types.

## 5 Conclusions

Ours is a small but homogeneous single-center study, which reports the real-life results that can be obtained through well-established and standardized methods available in numerous surgical pathology units around the world.

Immunohistochemical staining for p53 demonstrated excellent correlation with Sanger sequencing results, allowing patients to be divided into two subgroups with different prognoses. Overall, in our population high-grade serous carcinoma confirmed a severe prognosis with p53-null patients having an even more severe prognosis and who could benefit from differentiated therapeutic protocols with close follow-ups and more aggressive treatments.

Our results appear encouraging and could be the starting point for a larger, multi-center study with a numerosity permitting a multivariate analysis.

## Data availability statement

The original contributions presented in the study are included in the article/**Supplementary Material**. Further inquiries can be directed to the corresponding authors.

## Ethics statement

The studies involving humans were approved by Liguria Region, Genova, Italy (n127/2022-DB id12223). The studies were conducted in accordance with the local legislation and institutional requirements. The participants provided their written informed consent to participate in this study.

## Author contributions

Conceptualization, CB, VV, EM. Methodology, GL, AP. Software, MG, SP. Formal analysis, GL. Investigation, GC. Resources, VP. Data curation, GF. Writing-original draft preparation, CB. Writing-review and editing, MP. Visualization, CC and SM. Supervision and project administration, EM and VV. All authors contributed to the article and approved the submitted version.

## Supplementary material

Supplementary Figure 1 (200x) hematoxylin-eosin staining (up) and differences of p53 staining (down) between p53 wildtype mucinous carcinoma (left), HGSOC p53 null (center), and HGSOC p53 overexpressed (right).
