# Supplementary figures and images for: The fading guardian: clinical relevance of TP53 null mutation in high-grade serous ovarian cancers
Source: Front Immunol. 2023 Aug 23;14:1221605. doi: 10.3389/fimmu.2023.1221605 (PMC10480567; doi:10.3389/fimmu.2023.1221605)

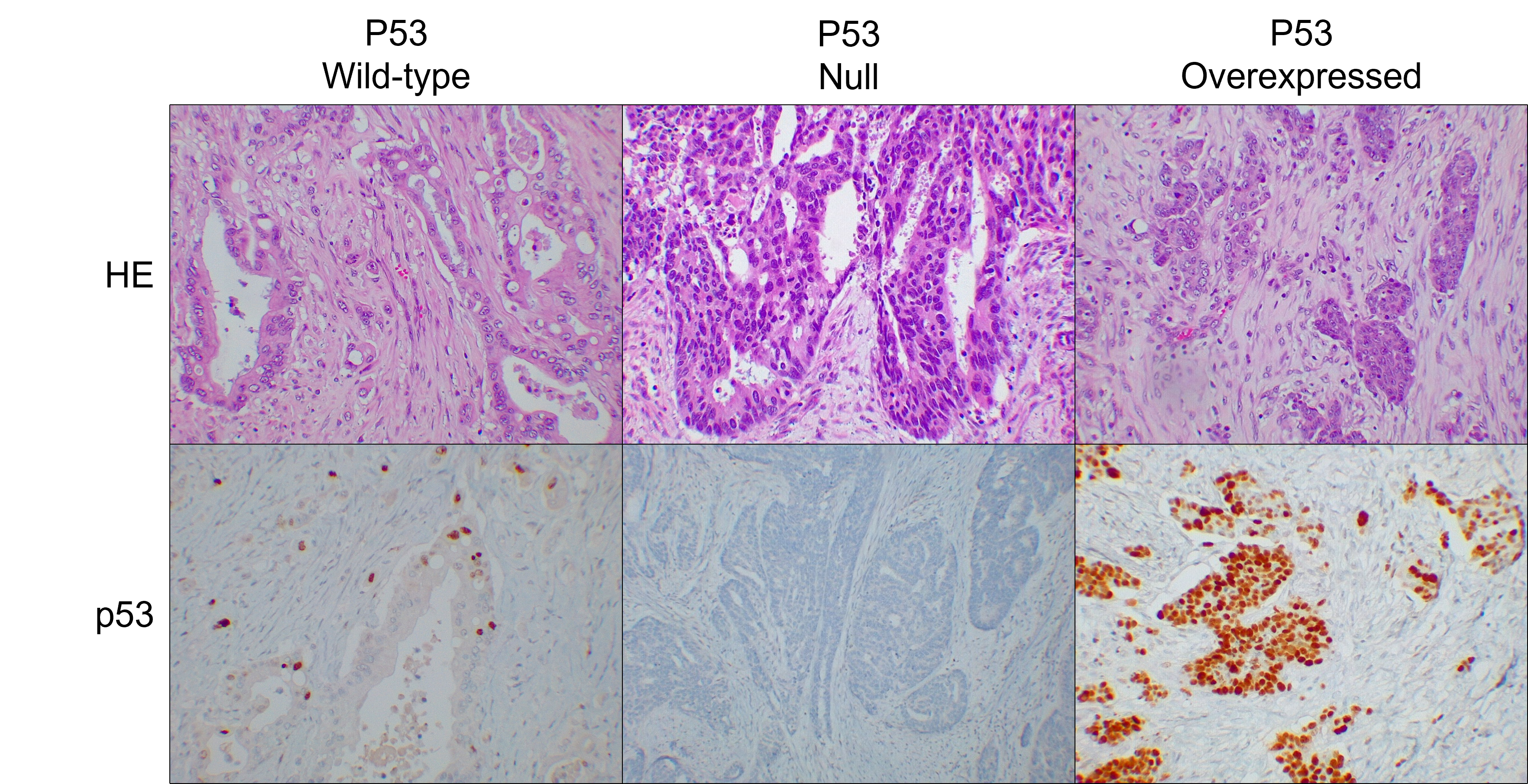

Supplement: Supplementary Figure 1 — (200x) hematoxylin-eosin staining (up) and differences of p53 staining (down) between p53 wildtype mucinous carcinoma (left), HGSOC p53 null (center), and HGSOC p53 overexpressed (right). [file Image_1.tif]
